# Combined Hyaluronic Acid and Collagen Injections for Perioral Rejuvenation: A Randomized Controlled Trial

**DOI:** 10.1111/jocd.71169

**Published:** 2026-08-30

**Authors:** Qing Liu, Chen Huang, Juan Zhang, Zhen Liang, Jiaxi Liu, Baoqiang Song

**Affiliations:** ^1^ Xian Bravou Medical Beauty Hospital Xi'an China; ^2^ Department of Plastic Surgery Xijing Hospital, Fourth Military Medical University Xi'an China; ^3^ Department of Dermatology The Second Affiliated Hospital of Kunming Medical University Kunming China

**Keywords:** collagen, hyaluronic acid, perioral rejuvenation

## Abstract

**Background:**

The perioral region, a critical facial aesthetic subunit, undergoes age‐related changes addressed primarily through dermal fillers. Optimal rejuvenation requires tailored approaches integrating anatomical precision, filler physicochemical properties, and standardized aging assessments.

**Objective:**

To prospectively compare hyaluronic acid (HA)‐collagen combination therapy against monotherapies for perioral rejuvenation using protocol‐guided aging evaluations and personalized strategies.

**Methods:**

Ninety female patients with perioral aging were randomized equally into three groups (Dec 2022–Dec 2023): Group A (HA alone), Group B (HA‐collagen), and Group C (collagen alone). Treatments were customized using baseline Comprehensive Perioral Aging Scale (CPAS) scores. Outcomes were assessed via CPAS and Global Aesthetic Improvement Scale (GAIS) posttreatment, at 3 months, and 6 months.

**Results:**

Group B demonstrated statistically superior improvements in CPAS and GAIS versus monotherapy groups at all intervals (*p* < 0.05). The combination regimen achieved optimal tissue support, lifting effects, and aesthetic outcomes, with sustained benefits at 6‐month follow‐up. Baseline characteristics showed no intergroup differences.

**Conclusion:**

Protocol‐guided HA‐collagen synergy produces enhanced perioral rejuvenation compared to single‐agent therapies, offering improved structural support and longevity of aesthetic corrections. This randomized trial validates personalized CPAS‐based combination approaches for age‐related perioral changes.

## 1 Introduction

The perioral region, a pivotal component of facial aesthetics, is highly prone to age‐related manifestations such as nasolabial folds, marionette lines, and perioral rhytides due to its dynamic functional demands and multifactorial influences [1]. Existing grading systems, which focus on isolated aging features (e.g., nasolabial fold severity or wrinkle classification), lack the capacity to holistically evaluate the composite aging process, complicating treatment strategies for this anatomically complex area. To address this gap, we developed the CPAS, a unified grading framework integrating multiple aging parameters, which has undergone preliminary clinical validation. Concurrently, advancements in nonsurgical rejuvenation have positioned injectable fillers as a cornerstone of anti‐aging therapies. HA, the most widely used filler, offers structural support and reversibility but carries risks of edema and migration in the perioral region [2]. Collagen‐based fillers, while mitigating these drawbacks, exhibit limited durability and mechanical stability [3]. This dichotomy prompted our hypothesis that synergistic combination therapy—leveraging HA's structural properties and collagen's natural integration—may optimize outcomes for heterogeneous aging patterns.

Guided by the CPAS, this prospective randomized controlled trial aims to validate the clinical utility of this novel scale while investigating the efficacy of combined HA and collagen injections. The dual objectives reflect a paradigm shift toward precision medicine in facial rejuvenation: first, transcending conventional single‐feature assessments to quantify global perioral aging severity, and second, addressing material‐specific limitations through strategic filler combinations. By tailoring injection protocols to CPAS‐derived anatomical profiles, this study bridges critical gaps in both assessment methodologies and multimodal therapeutic strategies, offering a framework for personalized, evidence‐based perioral rejuvenation.

## 2 Patients and Methods

This study was conducted in accordance with the Declaration of Helsinki and received approval from the institutional ethics committee of Xijing Hospital. All participants provided written informed consent prior to enrollment. Between December 2022 and December 2023, 90 patients meeting predefined criteria were recruited. Inclusion criteria comprised: (1) age > 20 years, (2) clinically evident perioral aging signs, and (3) voluntary participation with signed consent. Exclusion criteria included: (1) active perioral pathologies or chronic conditions contraindicating injections, (2) prior perioral treatments within the preceding 24 months, and (3) premature withdrawal from the study. Rigorous adherence to these criteria ensured cohort homogeneity and methodological integrity.

### 2.1 Patients Grouping

The cohort of 90 participants was randomly divided into three equal groups (*n* = 30 per group) using a computer‐generated randomization protocol: Group A received HA monotherapy, Group B underwent combined HA and collagen therapy (1:1 volume ratio), and Group C received collagen monotherapy. This triple‐arm design enabled direct comparison of material‐specific effects and potential synergies between the two filler types.

### 2.2 Comprehensive Perioral Aging Scale

The manifestations of perioral aging include skin photoaging, nasolabial fold wrinkles, melomental folds, perioral mounds, jawline contour deformities, lip fullness changes, and perioral lines. For each individual manifestation, grading was performed based on established clinical experience or validated scales from prior research. A comprehensive evaluation of perioral aging was subsequently conducted by integrating these assessments (Table.S1):

Skin Photoaging Scale: In 1996, Richard G. Glogau proposed a widely recognized classification for skin photoaging severity, which was adopted to evaluate perioral skin [4].

0: Smooth skin without wrinkles, pigmentation, or keratinization; 1: Mild perioral wrinkles, no pigmentation or keratinization; 2: Sparse dynamic wrinkles and faint pigmentation, with mild palpable keratinization and visible pores; 3: Static wrinkles, evident skin discoloration, pronounced pigmentation, visible keratinization, and telangiectasia; 4: Extensive wrinkles, marked pigmentation, enlarged pores, sallow skin tone, and precancerous or early neoplastic lesions.

Nasolabial Fold Wrinkle Scale: The scale by Lawrence Buchner et al. was utilized for precise grading [5]:

0: No wrinkles; 1: Shallow wrinkles; 2: Moderate wrinkles; 3: Deep wrinkles with defined edges; 4: Extremely deep wrinkles with multiple folds.

Melomental Folds Scale: The validated digital scale by Alastair Carruthers et al. [6] was applied:

0: No visible wrinkles [6]; 1: Shallow wrinkles with slight indentations; 2: Moderate wrinkles showing creases upon stretching; 3: Long, deep wrinkles; 4: Extremely deep wrinkles.

Perioral Mound Scale: The classification by Gu  et al. was employed [7]:

0: No perioral mounds; 1: Mounds appear only upon head lowering, absent in frontal or head‐raising positions; 2: Mounds visible in frontal or head lowering positions, absent when head‐raising; 3: Mounds present in all head positions; 4: Prominent mounds even during head‐raising positions.

Lip Fullness Scale: The Allergan Lip Fullness Scale, a reliable tool for clinical assessment [2], was used:

0: Full, vermilion‐rich lips; 1: Vermilion‐rich lips with lower lip elevation and moderate upper lip projection; 2: Moderate vermilion hue with mild lower lip elevation; 3: Mild vermilion hue, no elevation in either lip; 4: Minimal vermilion display, flattened lip contour.

Perioral Lines Scale: Based on Joel L. Cohen et al.'s standardized metrics for perioral lines at rest, oral commissures, and maximum contraction, a composite score was derived [8]:

0: No wrinkles; 1: Few, shallow wrinkles; 2: Sparse wrinkles of moderate depth; 3: Numerous wrinkles; 4: Numerous, deeply etched wrinkles.

Jawline Contour Scale: Following Yang et al.'s classification, jawline aging was graded [9]:

0: Defined jawline; 1: Clear jawline in upright position with fine wrinkles only upon head lowering positions; 2: Blunted jawline with fine wrinkles in upright position, resolving upon head‐raising positions; 3: Visible jawline laxity and coarse wrinkles persisting during head‐raising positions; 4: Severe laxity and deep wrinkles unaffected by head position.

Each subscale utilized a 0–4 scoring system, with higher scores indicating greater severity. These individual scales were integrated into a CPAS. Total scores determined global aging severity (Table S2): 0 (normal), 1–7 (mild), 8–22 (moderate), and 23–28 (severe). This framework enables systematic, multidimensional evaluation of perioral aging for targeted therapeutic planning.

### 2.3 Injection Technique

All injections were administered by a single physician. For Group A, Restylane Vital hyaluronic acid filler (Galderma, Lausanne, Switzerland) was used. Group C received Fillderm collagen‐based filler (Fiman, Beijing, China). For Group B, equal volumes of the aforementioned hyaluronic acid and collagen fillers were combined prior to injection. Injection techniques were tailored according to each patient's CPAS classification.

**FIGURE 1:**
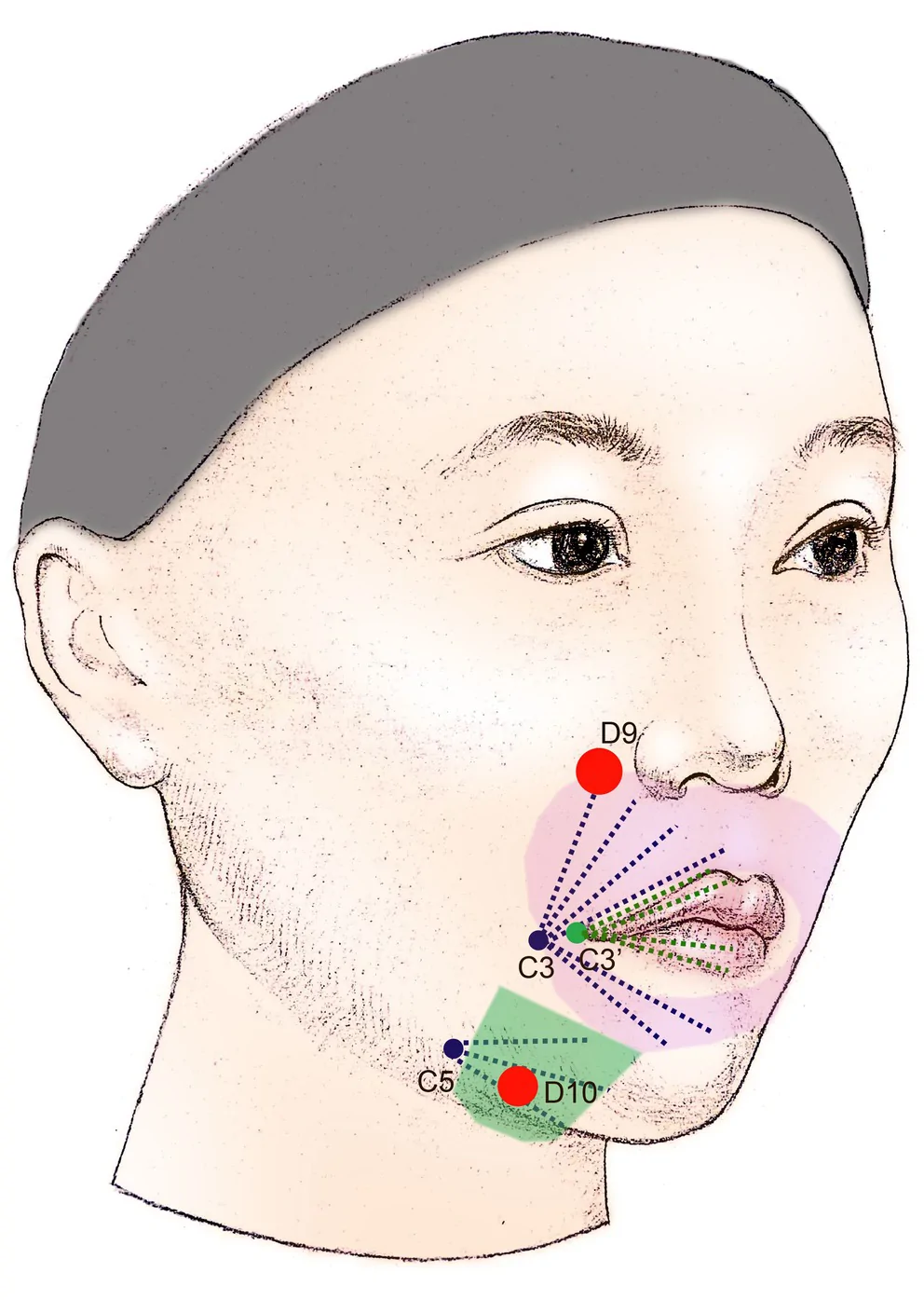
Injection points for mild perioral aging.

For the majority of patients with mild perioral aging, the following injection sites, tissue layers, and dosages were applied (Figure 1): Maxillary ligament and pyriform aperture space (D9): Deep nasolabial fold layer, 0.1–0.3 mL per side. Perioral region 1–1.5 cm lateral to the oral commissure (C3): Superficial nasolabial fat pad, 0.1–0.2 mL per side. 1–1.5 cm lateral to the oral axis (C3): Superficial fat pads of the upper and lower lips, 0.2–0.3 mL per side. 0.5 cm lateral to the oral commissure (C3′): Superficial fat pads of the upper and lower vermilion, 0.6–1 mL per side. Mandibular ligament area (D10): Mandibular ligament region, 0.1–0.2 mL per side. Central point of the perioral mound (C5): Perioral mound area, 0.2–0.5 mL per side. All dosages represent unilateral administration. The total injected volume for mild perioral aging patients typically ranged from 2 to 4 mL.

**FIGURE 2:**
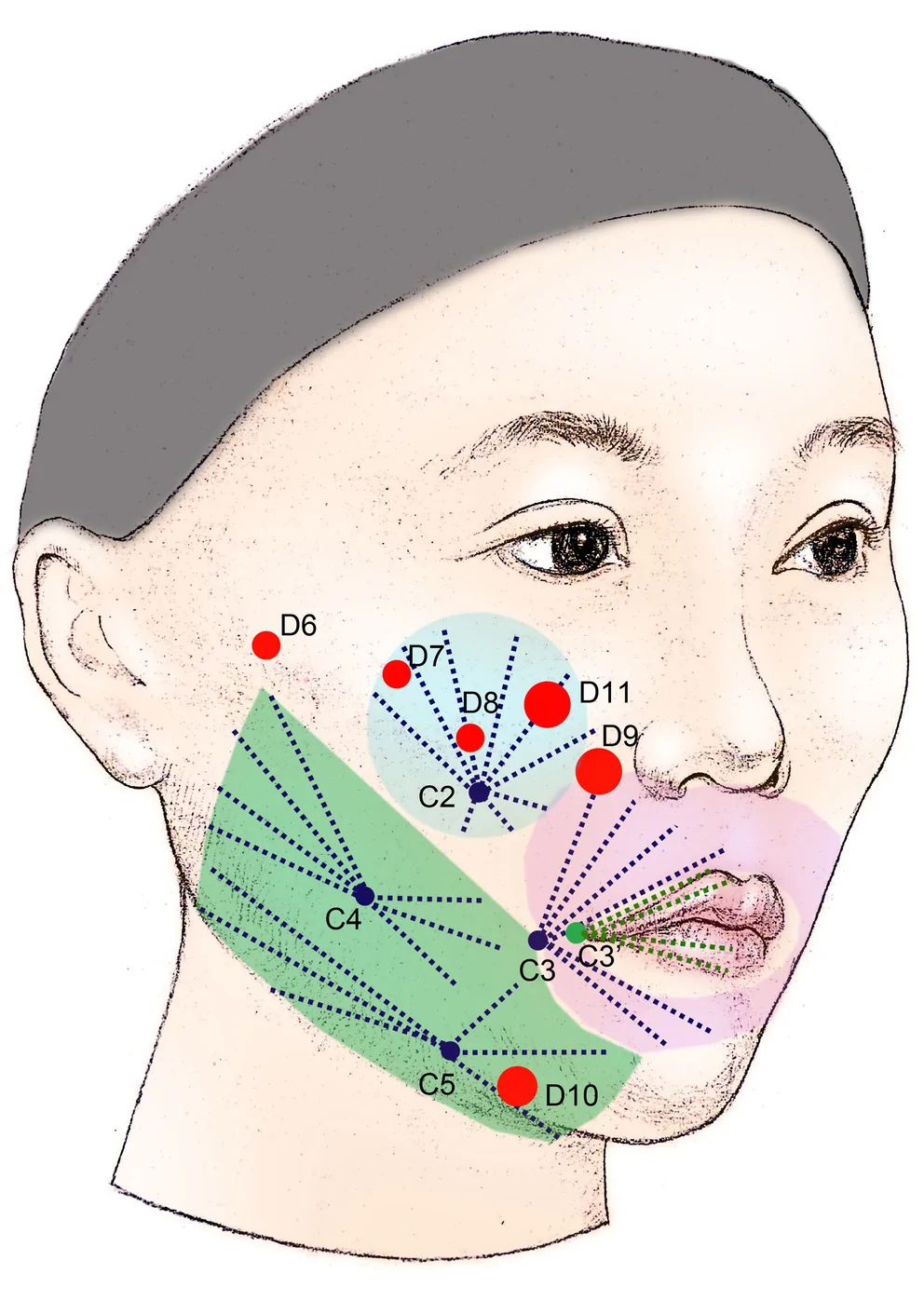
Injection points for moderate perioral aging.

For the majority of patients with moderate perioral aging, injections were administered at the following sites, tissue layers, and dosages (Figure 2): Maxillary ligament and pyriform aperture space (D9): Deep nasolabial fold layer, 0.2–0.3 mL per side. Perioral region 1–1.5 cm lateral to the oral commissure (C3): Superficial nasolabial fat pad, 0.2 mL per side.1–1.5 cm lateral to the oral axis (C3): Superficial fat pads of the upper and lower lips, 0.2–0.3 mL per side.0.5 cm lateral to the oral commissure (C3′): Superficial fat pads of the upper and lower vermilion, 1 mL per side. Mandibular ligament area (D10): Mandibular ligament region, 0.2–0.4 mL per side. Zygomatic ligament (D6), zygomatic retaining ligament (D7), zygomaticocutaneous ligament (D8), and deep buccal fat pad (D11): Injected into the zygomatic ligament complex and deep buccal fat compartment, with dosages of 0.1 mL, 0.1 mL, 0.1 mL, and 0.2–0.3 mL per side, respectively. Intersection of the lateral canthal vertical line and nasal base horizontal line (C2): Ogee line and superficial malar fat pad, 0.2–0.3 mL per side. Midpoint of the anterior masseter border (C4) and central perioral mound (C5): Injected into the mandibular contour and retrojugal depression (parotideomasseteric fascia region), 0.2–0.4 mL and 0.3–0.5 mL per side, respectively. All dosages represent unilateral administration. The total injected volume for moderate perioral aging patients typically ranged from 5 to 7 mL.

**FIGURE 3:**
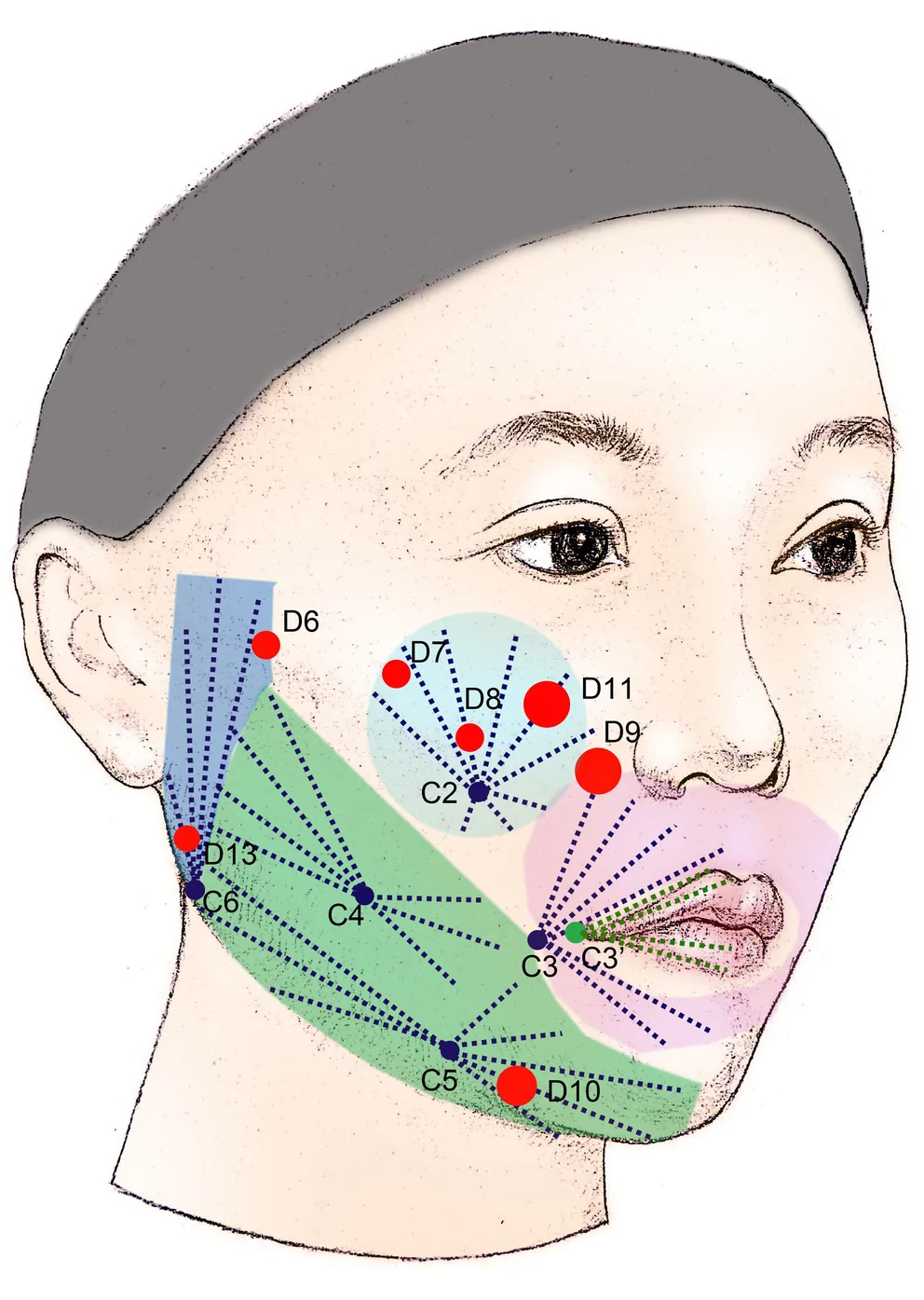
Injection points for severe perioral aging.

For the majority of patients with severe perioral aging, injections were administered at the following sites, tissue layers, and dosages (Figure 3): Maxillary ligament and pyriform aperture space (D9): Deep nasolabial fold layer, 0.3 mL per side. Perioral region 1–1.5 cm lateral to the oral commissure (C3): Superficial nasolabial fat pad, 0.2–0.3 mL per side.1–1.5 cm lateral to the oral axis (C3): Superficial fat pads of the upper and lower lips, 0.2–0.3 mL per side.0.5 cm lateral to the oral commissure (C3′): Superficial fat pads of the upper and lower vermilion, 1 mL per side. Mandibular ligament area (D10): Mandibular ligament region, 0.4–0.6 mL per side. Zygomatic ligament (D6), zygomatic retaining ligament (D7), zygomaticocutaneous ligament (D8), and deep buccal fat pad (D11): Injected into the zygomatic ligament complex and deep buccal fat compartment, with dosages of 0.1 mL, 0.1 mL, 0.2 mL, and 0.2–0.3 mL per side, respectively. Intersection of the lateral canthal vertical line and nasal base horizontal line (C2): Ogee line and superficial malar fat pad, 0.2–0.3 mL per side. Midpoint of the anterior masseter border (C4) and central perioral mound (C5): Injected into the mandibular contour and retrojugal depression (parotideomasseteric fascia region), 0.4–0.5 mL and 0.3–0.5 mL per side, respectively.

1 cm superior to the mandibular angle along the mandibular border (D13) and mandibular angle (C6): Injected into the preauricular platysmal region, 0.4–0.5 mL and 0.4–0.5 mL per side, respectively. All dosages represent unilateral administration. The total injected volume for severe perioral aging patients typically ranged from 8 to 10 mL (Table S3–S5).

### 2.4 Assessment Item

The evaluation metrics in our study included: (1) Baseline characteristics of the three patient groups, encompassing demographic data (age, gender), CPAS scores, and filler injection dosage; (2) Comparative analysis of CPAS changes between pre‐ and posttreatment status within individual patients, along with intergroup comparisons of CPAS variations among the three cohorts; (3) Intergroup comparisons of GAIS scores, employing the following grading criteria: −1 (deterioration), 0 (no change), 1 (mild improvement), 2 (significant improvement with residual adjustment potential), and 3 (complete improvement). The GAIS assessment comprised two distinct components: physician evaluation (conducted by a non‐injecting physician blinded to group allocation) and patient self‐assessment (the physician component of the GAIS was independently scored by a single experienced surgeon who was not involved in the injections and was fully blinded to treatment group allocation. Assessments were performed using standardized frontal and 45° oblique photographs taken under consistent lighting and positioning); (4) Systematic documentation of post‐procedural adverse events.

### 2.5 Statistic Analysis

Statistical analysis was conducted using SPSS 26.0 (IBM Statistics for Windows, IBM Corp., Armonk, NY, USA). Patient data were expressed as mean or median values based on distribution characteristics. Intergroup differences were analyzed through nonparametric methods: the Mann–Whitney U test for pairwise comparisons and the Nemenyi test for multiple group comparisons. A threshold of *p* < 0.05 was established to determine statistical significance.

## 3 Results

This study enrolled 90 female patients between December 2022 and December 2023, randomly allocated into groups A, B, and C. The mean ages across groups were 36.5, 37.4, and 39.2 years respectively. Baseline median CPAS scores pretreatment measured 13.5, 15.5, and 16.5 in the three groups. Therapeutic protocols comprised: hyaluronic acid monotherapy (Group A), combined hyaluronic acid with equivalent collagen (Group B), and collagen monotherapy (Group C). Mean filler dosages administered were 5.8 mL, 6.05 mL, and 6.75 mL respectively. No statistically significant differences were observed in these baseline parameters (detailed demographic characteristics presented in Table 1).

**TABLE 1:** Baseline data of patients.

Group	A	B	C	*p*
Age, year (mean ± SD)	36.50 ± 8.61	37.40 ± 8.53	39.20 ± 10.40	0.789
Gender	30F	30F	30F	\
Preoperative CPAS Median (P_25_, P_75_)	13.50 (5.75, 21.75)	15.50 (7.75, 22.50)	16.50 (9.25, 23.25)	0.842
Injection volume, ml (mean ± SD)	5.80 ± 2.18	6.05 ± 2.06	6.75 ± 2.10	0.573

Abbreviations: CPAS, Comprehensive perioral aging scale; F, Female; SD, Standard deviation.

Longitudinal CPAS evaluations revealed Group B demonstrated statistically superior score improvements compared to both Group A and C at all follow‐up intervals (immediately posttreatment, 3‐month, and 6‐month assessments). Notably, Group C exhibited minimal CPAS changes by the 6‐month endpoint (Table 2). GAIS assessments demonstrated differential outcomes between observer and patient evaluations: physicians identified significant intergroup differences favoring Group B at all timepoints, while patient self‐assessments only achieved statistical significance at the 6‐month evaluation (Table 3).

**TABLE 2:** Comprehensive Perioral Aging Scale (CPAS) scores.

Group	CPAS, Median (P_25_, P_75_)			*p*
	A	B	C	
M_1_‐M_0_	6.50 (2.75, 10.25)	13.00 (7.50, 18.25)	6.00 (3.75, 9.25)	0.047^a^
M_3_‐M_0_	5.00 (2.00, 8.00)	10.00 (6.00, 14.50)	4.00 (2.00, 7.00)	0.022^a^
M_6_‐M_0_	4.00 (1.00, 5.25)	7.00 (4.00, 11.00)	1.00 (1.00, 3.00)	0.005^a^

*Note:* M_0_: Preoperative. M_1_: Postoperative immediately. M_3_: Postoperative 3 months. M_6_: Postoperative 6 month. M_1_‐M_0_: Difference between posttreatment (immediate) and baseline CPAS scores. M_3_‐M_0_: Difference between 3‐month posttreatment and baseline CPAS scores. M_6_‐M_0_: Difference between 6‐month posttreatment and baseline CPAS scores.

^a^ Significant difference.

**TABLE 3:** Global aesthetic improvement scores (GAIS).

Group	GAIS, Median (P_25_, P_75_)			*p*
	A	B	C	
M1				
Observer score	2 (1.75, 3)	3 (2.75, 3)	2 (2, 3)	0.035^a^
Patient score	2 (2.5, 3)	2 (3, 3)	2 (2.5, 3)	0.907
M3				
Observer score	2 (1, 2)	2 (2, 2)	2 (1, 2)	0.049^a^
Patient score	2 (1, 2.25)	2 (1.75, 2.25)	1.5 (1, 2)	0.216
M6				
Observer score	1.5 (1, 2)	2 (1, 2)	1 (0.75, 1.25)	0.037^a^
Patient score	1 (1, 1)	1 (1, 2)	1 (0, 1)	0.018^a^

*Note:* M_1_: Postoperative immediately. M_3_: Postoperative 3 months. M_6_: Postoperative 6 month.

^a^ Significant difference.

Post‐procedural adverse events were systematically documented, with transient swelling (84.4%), bruising (72.2%), and injection‐site pain (66.7%) constituting the most frequent complications. Filler migration occurred in 3.3% of cases (Table 4).

**TABLE 4:** Adverse event posttreatment.

Adverse event	Group A	Group B	Group C	*p*
Swelling	4	2	0	0.127
Bruising	2	1	3	0.565
Pain	0	1	1	0.598
Pigmentation	0	0	0	1
Displacement	1	1	0	0.613

## 4 Cases Report

### 4.1 Cases 1

**FIGURE 4:**
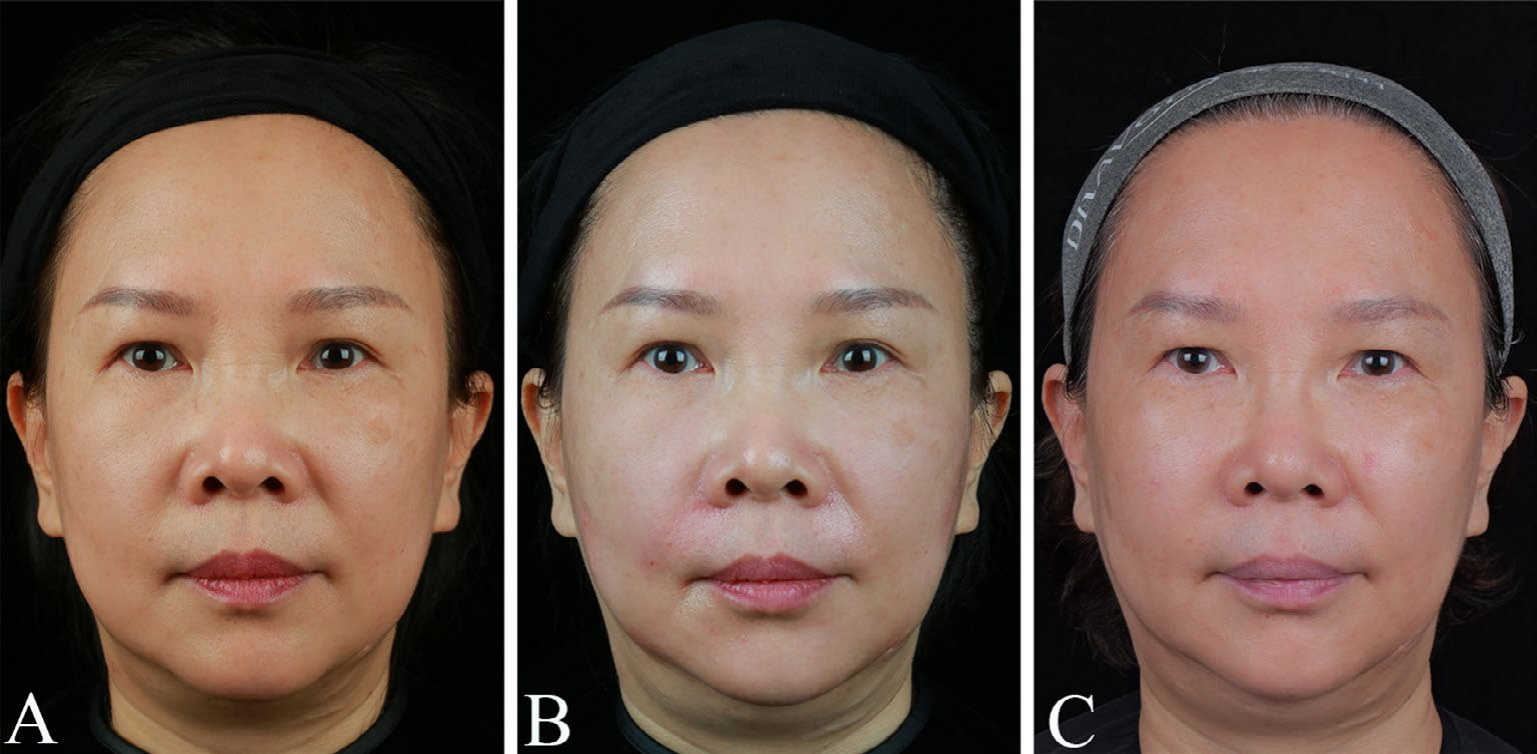
Posttreatment outcomes in the patient demonstrating satisfactory perioral rejuvenation immediately and at 3 months postinjection, with partial re‐emergence of aging characteristics in specific perioral regions observed at the 6‐month follow‐up. A: Before injection; B: Immediate post‐injection effects; C: 6 months after injection.

A 52‐year‐old female patient presented to our institution seeking treatment for self‐perceived perioral aging. Comprehensive CPAS evaluation demonstrated the following parameters: perioral skin aging score of 3, nasolabial fold score of 2, oral commissure festoons score of 2, lip volume score of 2, perioral rhytides score of 2, and mandibular contour score of 4, yielding a total score of 15 indicative of moderate perioral aging. Collagen‐based filler injections were administered according to the following protocol: D6, D7, D8 injection points: 0.1 mL per site (unilateral); D9, D10, C3, C3′ injection points: 0.3 mL per site (unilateral); C4, C5 injection points: 1.0 mL per site (unilateral); The total administered volume reached 7 mL bilaterally. Immediate posttreatment assessment revealed significant improvement in perioral rejuvenation. However, follow‐up at 6 months demonstrated partial recurrence of aging characteristics in specific perioral regions (Figure 4).

### 4.2 Cases 2

**FIGURE 5:**
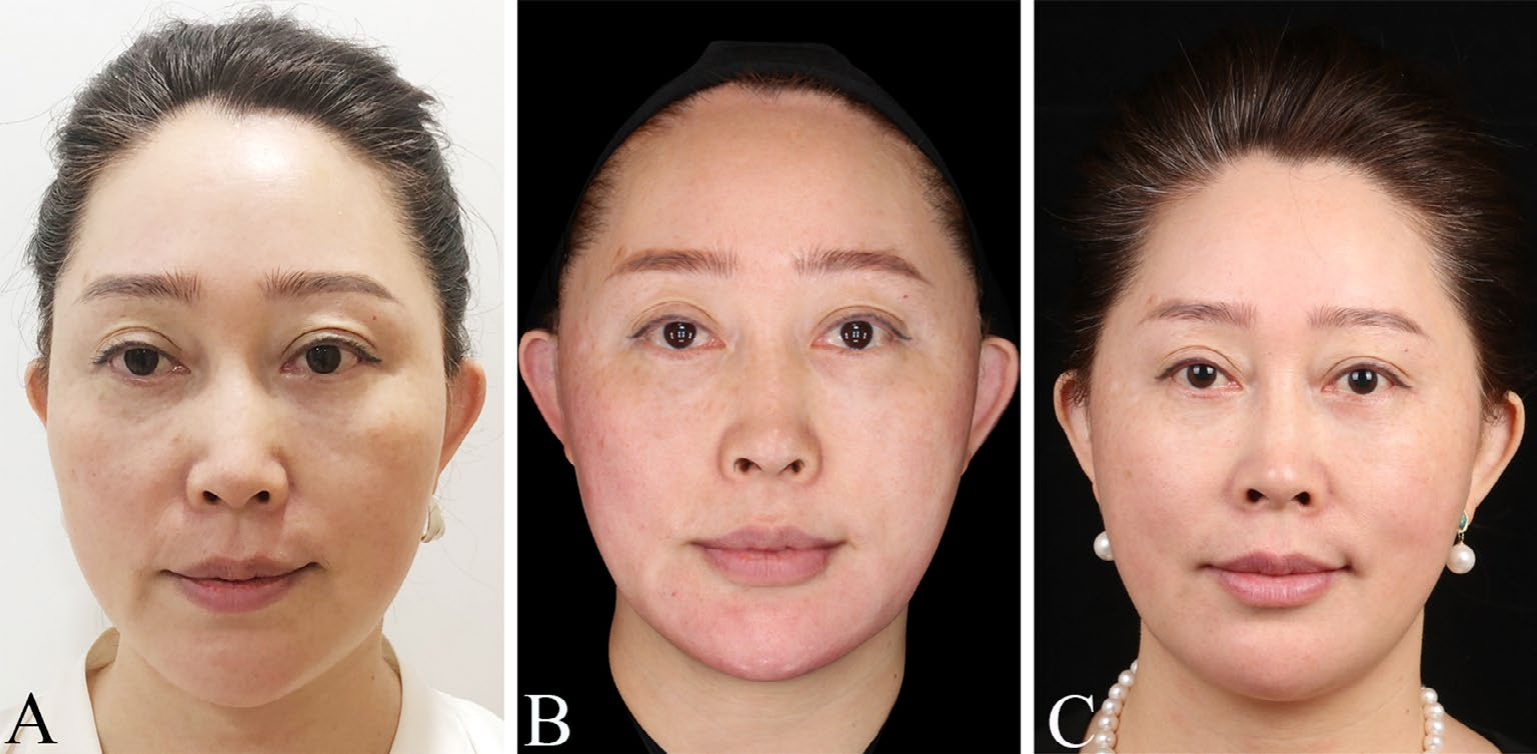
Patient exhibited optimal perioral rejuvenation immediately posttreatment, with sustained therapeutic efficacy maintained at the 6‐month follow‐up assessment. A: Before injection; B: Immediate post‐injection effects; C: 6 months after injection.

A 47‐year‐old female patient presented with moderate perioral aging, as evidenced by a comprehensive CPAS evaluation: perioral skin aging score of 2, nasolabial fold score of 2, oral commissure festoons score of 2, lip volume score of 1, perioral rhytides score of 1, and mandibular contour score of 2, yielding a total score of 10. A combined collagen and hyaluronic acid filler protocol was administered with the following specifications: D6, D7, D8 injection points: 0.1 mL per side bilaterally; D11, C2 injection points: 0.3 mL per side bilaterally; D9, D10 injection points: 0.2 mL (left) and 0.3 mL (right) respectively; C3, C3′ injection points: 0.3 mL per side bilaterally; C4 injection point: 0.9 mL (left) and 1.0 mL (right); C5 injection point: 0.8 mL (left) and 1.0 mL (right). The total administered volume reached 8 mL bilaterally. Immediate posttreatment evaluation demonstrated marked perioral rejuvenation, with sustained efficacy observed during follow‐up assessments. The therapeutic outcome exhibited prolonged durability, achieving high patient satisfaction (Figure 5).

## 5 Discussion

Perioral aging represents a complex multifactorial process involving synergistic biological mechanisms [10, 11]. Primarily, posterior displacement and remodeling of the craniofacial skeleton occur with advancing age, characterized by progressive bone resorption and structural alterations that disrupt the spatial relationships of overlying soft tissues, adipose compartments, and muscular architecture. Secondly, ligamentous degeneration contributes to soft tissue laxity, manifesting clinically as perioral skin redundancy, oral commissure ptosis, and deepening of nasolabial folds. Furthermore, chrono‐gravitational effects induce compartment‐specific adipose changes: atrophy of deep fat compartments coupled with inferior migration of superficial fat pads. Muscular senescence plays a pivotal role through age‐related muscular atrophy and positional descent, exacerbating cutaneous and subcutaneous tissue laxity. The superficial musculoaponeurotic system (SMAS) demonstrates compromised structural integrity due to collagen depletion and fat pad displacement, resulting in loss of vertical support for overlying tissues. Ultimately, intrinsic cutaneous aging mechanisms—including collagen fiber fragmentation, reduced type I collagen synthesis, diminished hyaluronic acid content, impaired water retention capacity of the extracellular matrix, elastic fiber degradation, and sebum production decline—collectively exacerbate perioral aging manifestations. This multifactorial interplay necessitates holistic evaluation of perioral aging severity and implementation of comprehensive therapeutic strategies addressing both anatomical substrates and biochemical alterations.

For decades, hyaluronic acid has remained the principal filler for perioral rejuvenation [12]. However, it exhibits drawbacks such as postinjection edema and susceptibility to migration, particularly in the highly dynamic perioral region [13]. In recent years, collagen has gained increasing application in treating perioral aging. Nevertheless, compared to hyaluronic acid, collagen demonstrates inferior tissue support, lifting capacity, and faster metabolic turnover—a conclusion supported by the substantial decline in GAIS scores and CPAS improvements observed in Group C patients at 6‐month posttreatment in our study. This evidence prompted our hypothesis that combining hyaluronic acid with collagen might synergistically leverage their respective advantages while mitigating individual limitations. Our results demonstrated that Group B patients (receiving combination therapy) achieved optimal CPAS and GAIS scores with statistically significant differences compared to other groups, both immediately after treatment and at 6‐month follow‐up. These findings confirm that the combined use of hyaluronic acid and collagen enhances soft tissue support, lifting efficacy, and filler retention duration compared to single filler applications, thereby demonstrating excellent therapeutic outcomes.

Additionally, a discrepancy was observed between physician‐assessed and patient self‐reported GAIS scores. The blinded physician detected statistically significant superiority of the combination therapy at all‐time points, whereas patients reported significance only at the 6‐month follow‐up. This pattern, frequently noted in aesthetic medicine trials, may be attributable to several factors. Physicians, as trained specialists, are more sensitive to subtle early anatomical improvements visible on standardized photographs. In contrast, patients often maintain higher aesthetic expectations and may be less perceptive of minor changes. Transient post‐procedural edema or tissue adaptation in the dynamic perioral region could also differentially affect immediate self‐perception. The alignment of patient self‐assessment with physician evaluation at 6 months nonetheless underscores the sustained clinical benefit and durability of the HA‐collagen combination. We acknowledge that both clinician‐rated and patient‐reported aesthetic scales carry inherent subjectivity and that placebo effects may influence self‐assessment in cosmetic procedures.

This study has several limitations. First, GAIS scoring by physicians was performed by a single blinded independent evaluator. Although strict blinding and standardized photography were employed, the single‐rater design prevented calculation of inter‐rater reliability (e.g., Cohen's kappa). This may introduce potential observer bias. Future multicenter studies should incorporate multiple independent blinded raters. Second, the study relied on validated subjective scales (CPAS and GAIS). While clinically relevant, these scales inherently contain subjectivity. Objective instrumental assessments (e.g., three‐dimensional volumetric analysis or skin texture quantification) were not available during the study period. We recommend that subsequent research integrate such objective measures to complement visual scoring. Finally, the 6‐month follow‐up duration, while standard for many filler studies, could be extended to better characterize long‐term outcomes.

## 6 Conclusion

Building upon personalized CPAS grading and injection protocols, this prospective, controlled, randomized clinical study demonstrates that the combined application of hyaluronic acid and collagen achieves superior soft tissue support and lifting effects in perioral rejuvenation. The combination therapy significantly prolongs filler retention duration compared to monotherapy approaches, thereby delivering clinically optimized therapeutic outcomes.

## Author Contributions

Q.L. and C.H. performed the research. B.S. and J.L. designed the research study. Z.L. and J.Z. analyzed the data. Q.L. and C.H. wrote the paper.

## Funding

The authors have nothing to report.

## Disclosure

This randomized clinical trial adheres to the CONSORT guidelines and indicates this in the article.

## Ethics Statement

We received approval from the ethics committee, and the approval number is KY202423259‐F‐1 and the study complied with the Helsinki Declaration.

## Consent

All patients consent to the publication of their photo in relevant medical publications for educational and research purposes.

## Conflicts of Interest

The authors declare no conflicts of interest.

## Supporting information


**Table S1:** Comprehensive Perioral Aging Scale.
**Table S2:** CPAS scores and corresponding classifications.
**Table S3:** Injection sites and doses for mild perioral aging.
**Table S4:** Injection sites and doses for moderate perioral aging.
**Table S5:** Injection sites and doses for severe perioral aging.

## Data Availability

The data that support the findings of this study are available from the corresponding author upon reasonable request.
