# Supplementary material for: Combined Hyaluronic Acid and Collagen Injections for Perioral Rejuvenation: A Randomized Controlled Trial
Source: J Cosmet Dermatol. 2026 Aug 30;25(9):e71169. doi: 10.1111/jocd.71169 (PMC13526530; doi:10.1111/jocd.71169)
Supplement: Supplementary file 1 [file JOCD-25-e71169-s001.docx]

**Supplementary table.1**  Comprehensive Perioral Aging Scale

| **Grade** | **Skin Photoaging** | **Nasolabial Fold** | **Melomental Folds** | **Perioral Mound** | **Lip Fullness** | **Perioral Lines** | **Jawline Contour** |
| --- | --- | --- | --- | --- | --- | --- | --- |
| **0** | Smooth skin, no wrinkles, no discoloration or keratinization | No wrinkles | No noticeable wrinkles | No oral commissure pouch | full and well-colored | No perioral wrinkles | clear jawline contour |
| **1** | Slight wrinkles around the mouth, no discoloration or keratinization | Shallow wrinkles | Shallow wrinkles with slight indentations | No tissue sagging when viewed at eye level or upward, but a sagging pouch appears when the head is lowered | well-colored, with the lower lip slightly elevated and the upper lip moderately elevated | Few and shallow perioral wrinkles | jawline is clear when the patient is seated and looking straight ahead, with no wrinkles on the skin; however, fine and shallow wrinkles appear when the head is lowered |
| **2** | Mild dynamic wrinkles and faint discoloration, with slight keratinization and noticeable pores | Moderate wrinkles | Moderate wrinkles, with folds visible when stretched | Oral commissure pouch visible when viewed at eye level or with the head lowered, but disappears when looking upward | moderately colored, with the lower lip slightly elevated. | A limited number of wrinkles with moderate depth | less prominent when the patient is seated and looking straight ahead, with fine and shallow wrinkles present; these wrinkles disappear when the head is tilted back |
| **3** | Static wrinkles around the mouth, uneven skin color, noticeable age spots, visible keratinization, and capillary dilation | Deep wrinkles with well-defined edges | Long, deep wrinkles | Oral commissure pouch appears when viewed at eye level, with the head lowered, and when looking upward | slight color, with no elevation in either the upper or lower lip | Numerous wrinkles | noticeable submental fullness when seated and looking straight ahead, with coarse and deep wrinkles on the skin; these wrinkles do not disappear when the head is tilted back. |
| **4** | Extensive wrinkles around the mouth, numerous age spots, enlarged pores, overall yellowish skin tone, mixed precancerous lesions, and early skin tumors | Very deep wrinkles with multiple folds | Extremely deep wrinkles | Oral commissure pouch is clearly visible even when the patient is looking upward | minimally visible, with the lip contour appearing almost flat and lacking curvature | Large number of very deep wrinkles | significant submental fullness when seated and looking straight ahead, with coarse and deep wrinkles on the skin that remain very prominent even when tilting the head back |

**Supplementary table.2** CPAS scores and corresponding classifications

| CPAS scores | Grade of perioral aging |
| --- | --- |
| 0 | normal |
| 1-7 | mild |
| 8-22 | moderate |
| 23-28 | severe |

**Supplementary table.3** Injection sites and doses for mild perioral aging

|  | **Region and level** | **Point** | **Volume** |
| --- | --- | --- | --- |
| **Part 2** | Deep nasolabial fold | D9:Maxillary ligament and pyriform aperture space. | 0.1-0.3ml |
|  | Superficial nasolabial fat pad. | C3:1-1.5 cm lateral to the oral axis. | 0.1-0.2ml |
|  | Mandibular ligament region. | D10:Mandibular ligament. | 0.1-0.2ml |
|  | Superficial fat pads of the upper and lower lips | C3:1-1.5 cm external to the oral axis. | 0.2-0.3ml |
|  |  | C3:0.5 cm lateral to the oral commissure (upper and lower vermilion) | 0.3-0.5ml |
|  | Oral commissure pouch region. | C5:Center point of the buccal pouch. | 0.2-0.5ml |

The D point injection uses a 30G, 13mm sharp needle to reach the periosteum, injecting from top to bottom and from outside to inside. The C injection point employs a 25G or 27G blunt needle to inject into the deep or superficial fat layer, starting with the deep layer and progressing to the superficial layer. The injection is performed in a fan-shaped pattern with point-by-point retreat, with 4-5 injection points per line, each receiving 0.01-0.02 ml. The aforementioned dosages represent the unilateral injection amount, with a total injection volume of 2-4 ml.

**Supplementary table.4** Injection sites and doses for moderate perioral aging

|  | **Region and level** | **Point** | **Volume** |
| --- | --- | --- | --- |
| **Part 1** | Zygomatic ligament and deep fat pads of the cheek. | D6: Zygomatic arch ligament  D7:Zygomaticomaxillary igament  D8: Zygomatic skin ligament  D11: Deep fat pads of the cheek | 0.1ml  0.1ml  0.1ml  0.2-0.3ml |
|  | Ogee line and superficial fat pads of the malar region. | C2: Intersection of the lateral canthus vertical line and the horizontal line at the base of the ala nasi. | 0.2-0.3ml |
|  | Deep layer of the nasolabial fold. | D9: Gap between the maxillary ligament and the piriform aperture. | 0.2-0.3ml |
| **Part 2** | Superficial fat pad of the nasolabial fold. | C3: 1-1.5 cm lateral to the oral axis. | 0.2ml |
|  | Mandibular ligament region | D10: Mandibular ligament. | 0.2-0.4ml |
|  | Superficial fat pads of the upper and lower lips. | C3: 1-1.5 cm external to the oral axis. | 0.2-0.3ml |
|  |  | C3: 0.5 cm lateral to the oral commissure (upper and lower lips). | 0.5ml |
| **Part 3** | The contour of the lower jaw and the posterior cheek depression (parotid masseteric fascia region). | C4: Mid-portion of the anterior border of the masseter muscle. | 0.2-0.4ml |
|  |  | C5: Center point of the buccal pouch at the oral commissure. | 0.3-0.5ml |

At the D Point, a 30G, 13mm sharp needle is employed to inject into the periosteum, with the injection performed from top to bottom and from outside to inside. At the C Point, a 25G or 27G blunt needle is used to inject into the deep or superficial fat layer, beginning with the deep layer and progressing to the superficial layer. The injection follows a fan-shaped distribution, utilizing a spot-back technique. Each line consists of 4-5 injection points, with each point receiving 0.01-0.02 ml. The specified dosages refer to unilateral injection, resulting in a total injection volume of 5.0-7.0 ml.

**Supplementary table.5** Injection sites and doses for severe perioral aging

|  | **Region and level** | **Point** | **Volume** |
| --- | --- | --- | --- |
| **Part 1** | Zygomatic ligament and deep fat pads of the cheek. | D6: Zygomatic arch ligament  D7:Zygomaticomaxillary igament  D8: Zygomatic skin ligament  D11: Deep fat pads of the cheek | 0.1ml  0.1ml  0.2ml  0.2-0.3ml |
|  | Ogee line and superficial fat pads of the malar region | C2: Intersection of the lateral canthus vertical line and the horizontal line at the base of the ala nasi. | 0.2-0.3ml |
|  | Deep layer of the nasolabial fold. | D9: Gap between the maxillary ligament and the piriform aperture. | 0.3ml |
| **Part 2** | Superficial fat pad of the nasolabial fold. | C3: 1-1.5 cm lateral to the oral axis. | 0.2-0.3ml |
|  | Mandibular ligament region | D10: Mandibular ligament. | 0.4-0.6ml |
|  | Superficial fat pads of the upper and lower lips. | C3: 1-1.5 cm external to the oral axis. | 0.3ml |
|  |  | C3: 0.5 cm lateral to the oral commissure (upper and lower lips). | 0.5ml |
| **Part 3** | The contour of the lower jaw and the posterior cheek depression (parotid masseteric fascia region). | C4: Mid-portion of the anterior border of the masseter muscle. | 0.4-0.5ml |
|  |  | C5: Center point of the buccal pouch at the oral commissure. | 0.3-0.5ml |
| **Part 4** | Anterior region of the sternocleidomastoid muscle. | D13: 1 cm below the mandibular angle from the lower border of the mandible | 0.4-0.5ml |
|  |  | C6: Mandibular angle | 0.4-0.5ml |

At the D Point, a 30G, 13mm sharp needle is used to inject into the periosteum, with the injection administered from top to bottom and from outside to inside. At the C Point, a 25G or 27G blunt needle is employed to inject into the deep or superficial fat layer, starting with the deep layer and followed by the superficial layer. A fan-shaped distribution and spot-back technique are used, with each line consisting of 4-5 injection points, each receiving 0.01-0.02 ml. The specified dosages refer to unilateral injection, resulting in a total injection volume of 8-10 ml.
